# The value of the neutrophil-lymphocyte count ratio in the diagnosis of sepsis in patients admitted to the Intensive Care Unit: A retrospective cohort study

**DOI:** 10.1371/journal.pone.0212861

**Published:** 2019-02-27

**Authors:** Kim Westerdijk, Koen S. Simons, Marissa Zegers, Peter C. Wever, Peter Pickkers, Cornelis P. C. de Jager

**Affiliations:** 1 Department of Intensive Care Medicine, Jeroen Bosch Hospital, ’s-Hertogenbosch, the Netherlands; 2 Department of Medical Microbiology and Infection Control, Jeroen Bosch Hospital, ’s-Hertogenbosch, the Netherlands; 3 Department of Intensive Care Medicine, Radboud University Nijmegen Medical Centre, Nijmegen, the Netherlands; San Gerardo Hospital, ITALY

## Abstract

**Background:**

Early diagnosis and treatment has proven to be of utmost importance in the outcome of sepsis patients. We compared the accuracy of the neutrophil-lymphocyte count ratio (NLCR) to conventional inflammatory markers in patients admitted to the Intensive Care Unit (ICU).

**Methods:**

We performed a retrospective cohort study consisting of 276 ICU patients with sepsis and 388 ICU patients without sepsis. We compared the NLCR as well as C-reactive protein (CRP) level, procalcitonin (PCT) level, white blood cell (WBC) count, neutrophil count and lymphocyte count on ICU admission between sepsis and non-sepsis ICU patients. To evaluate the sensitivity and specificity, we constructed receiver operating characteristics (ROC) curves.

**Results:**

Significant differences in NLCR values were observed between sepsis and non-sepsis patients (15.3 [10.8–38.2] (median [interquartile range] vs. 9.3 [6.2–14.5]; P<0.001), as well as for CRP level, PCT level and lymphocyte count. The area under the ROC curve (AUROC) of the NLCR was 0.66 (95%CI = 0.62–0.71). AUROC was significantly higher for CRP and PCT level with AUROC’s of 0.89 (95%CI 0.87–0.92) and 0.88 (95%CI 0.86–0.91) respectively.

**Conclusions:**

The NLCR is less suitable than conventional inflammatory markers CRP and PCT to detect the presence of sepsis in ICU patients.

**Trial registration:**

ClinicalTrials.gov NCT01274819.

## Introduction

Sepsis is a clinical syndrome that complicates severe infection. It is characterized by the cardinal signs of inflammation (vasodilation, leukocyte accumulation, increased vascular permeability) occuring in tissues that are remote from the site of infection. Sepsis is associated with a high mortality rate, especially in patients that need treatment in the Intensive Care Unit (ICU) [1]. In order to be able to initiate treatment in an early stage and improve prognosis of patients with sepsis, early and accurate diagnosis of sepsis is of utmost importance.

Diagnosing sepsis in severely ill patients remains a challenging task. In addition to the medical history and physical examination, laboratory markers of infection and inflammation play a major role in the final diagnosis. Currently, white blood cell (WBC) count, C-reactive protein (CRP) and procalcitonin (PCT) are commonly used to detect sepsis. However, increased levels of CRP can be found in various inflammatory conditions and, therefore, is of limited value in distinguishing infection from other causes of inflammation [2–4]. Studies concerning PCT as a diagnostic tool for differentiating sepsis from systemic inflammatory response syndrome (SIRS) show conflicting results due to heterogeneity of study populations [5–7].

The physiological immune response to infection and other stressful events is characterized by an increase in neutrophil count and a decrease in lymphocyte count. The increase in neutrophil count results from reduced apoptosis of neutrophils and rapid mobilization of neutrophils from a marginated pool within the bone marrow [1, 8, 9]. The lymphocyte count is decreased by migration of activated lymphocytes to inflammatory tissues and by increased apoptosis of lymphocytes [8, 10]. In 2001, Zahorec introduced the neutrophil-lymphocyte count ratio (NLCR) as a simple, rapid and cheap parameter of inflammation and stress in critically ill patients [11]. More recently, the predictive value of the NLCR in patients with suspected bacteremia in the Emergency Department (ED) and the association between the NLCR and both short- and long-term outcome in critically ill patients was described [12–14].

However, studies on the predictive value of the NLCR in diagnosing sepsis in ICU patients are lacking. Therefore, we performed the current study to determine whether the NLCR can be used to accurately establish a diagnosis of sepsis in ICU patients in comparison to WBC count, neutrophil count, lymphocyte count, CRP and PCT. Furthermore, we investigated the association between the NLCR and the duration of ICU stay, the duration of hospitalization, ICU mortality, in-hospital mortality and 6-month mortality.

## Materials and methods

### Patients

The current study was performed on data available from a previous randomized controlled trial evaluating effects of light therapy on the incidence and duration of ICU-acquired delirium (NCT01274819) [15]. Informed consent was obtained from all patients and the study was approved by the local medical ethical committee (METOPP, Medisch-Ethische Toetsing Onderzoek Patiënten en Proefpersonen, this name has been changed in METC (Medisch Ethische Toetsing Commissie) Brabant in January 1^st^ 2014, registration number M392 NL 34780.028.10, METOPP, Tilburg, The Netherlands). The study cohort consisted of 734 patients admitted to the ICU of the Jeroen Bosch Hospital in ‘s- Hertogenbosch between 2011 and 2013. Patients were admitted to the ICU when they had manifest or imminent organ failure, especially circulatory, respiratory or renal failure. The need for ICU admission was determined by the intensivist in collaboratrion with the treating doctor. No specific groups were excluded a priori. Details of the in- and exclusion criteria of this study can be found in the Supporting Information (S1 Text). Of importance is the fact that no patients were excluded due to untreatable severe sepsis. In the current study, patients with a known or probable immune deficiency were excluded, including patients with a haematological disease, patients treated with chemotherapy or immunosuppressive therapy including glucocorticoids. Finally, patients who had been admitted to an ICU elsewhere before admission to the ICU of the Jeroen Bosch Hospital were also excluded.

Patients were divided into two groups depending on the presence or absence of sepsis. Sepsis was defined as the presence of two or more SIRS criteria (body temperature of more than 38°C or less than 36°C, heart rate of more than 90 beats per minute, respiratory rate of more than 20 breaths per minute, and WBC count below 4*10^9^/L or above 12*10^9^/L) and the presence of infection, confirmed by radiological or microbiological investigation. Patients with SIRS and high clinical suspicion of infection as determined by the treating physician, in the absence of confirmation of infection, were considered as having sepsis as well.

Of all patients, baseline clinical characteristics, as well as data concerning confirmation of infection, detected by microbiological investigation (positive cultures, serology or polymerase chain reaction (PCR)), radiological investigation (e.g. infiltrate on chest X-ray, abscess on CT) or during procedures (e.g. surgical procedure, percutaneous biliary drainage) within 3 days after admission to the ICU as well as previous antibiotic use were collected from patient records.

### Microbiology

Of all patients with SIRS and suspected infection, bacterial culture results were reviewed of both blood and other specimens such as urine, sputum, abdominal fluid, bile or feces. All bacterial isolates were identified by standard microbiologic procedures. Microbiological investigation also included urine immunochromatographic antigen detection tests, standard PCR techniques and serum enzyme-linked immunosorbent assays (ELISA) for antibody detection.

### Inflammatory markers

Measurements of CRP, PCT, WBC count, neutrophil count and lymphocyte count were performed on blood samples collected at admission to the ICU or, if specific markers were not determined directly on ICU admission, on blood samples taken within 6 hours prior to or after ICU admission. CRP levels were measured with a fully automated enzyme-linked immunoassay using an Aeroset 2.0 analyzer (Abbott Diagnostics, Santa Clara, CA, USA). PCT levels were measured using a commercially available sensitive immunoluminometric assay (LIA sensitive, Brahms AG, Henningsdorf, Germany). WBC count, neutrophil count and lymphocyte count were determined on a Sysmex XE-2100 haematology analyzer (Sysmex Corporation, Kobe, Japan). From September 2012 onwards, CRP levels and PCT levels were measured using a Dimension Vista 1500 analyzer (Siemens Healthcare, the Hague, Netherlands) and WBC count, neutrophil count and lymphocyte count using an Advia 2120i analyzer (Siemens Healthcare, the Hague, Netherlands). The NLCR was calculated by dividing the neutrophil count through the lymphocyte count (normal value 2–4). A PCT level above 100 ng/ml or below 0.02 ng/ml or a CRP level below 6 mg/l or below 3 mg/l (measurement limits) were considered as 101 ng/ml, 0.019 ng/ml, 5 mg/l and 2 mg/l, respectively.

### Outcome

The primary outcome in this study is the association between the NLCR and the presence of sepsis. Since previous studies focused on diagnosing bacteremia instead of sepsis, the association between the NLCR and the presence of bacteremia in patients with sepsis was also determined. Secondary outcome measures were the association between the NLCR and the duration of ICU stay, the duration of hospitalization, ICU mortality, in-hospital mortality and 6-month mortality. Also, the relation of NLCR and disease severity (APACHE II, SOFA) and site of infection were determined.

### Statistical analysis

Statistical analysis was performed using SPSS version 22. Baseline characteristics were compared using the chi-square test or Fisher exact test for categorical variables and the Student’s t-test for continuous variables. The inflammatory markers were tested for normality using the Kolmogorov-Smirnov test. Since none of the inflammatory markers were normally distributed, the Mann-Whitney U test was used to compare medians across the sepsis and non-sepsis group. Subgroup analysis was performed in the sepsis group to compare the medians across patients with and without bacteremia, respectively.

To evaluate the sensitivity and specificity of the NLCR in detecting sepsis, receiver operating characteristics (ROC) curves were constructed for the NLCR as well as CRP, PCT, WBC count, neutrophil count and lymphocyte count. ROC curves were also constructed to evaluate the sensitivity and specificity of the inflammatory markers in diagnosing bacteremia in patients with sepsis. To compare the area under the ROC curves of the individual inflammation parameters, the method described by Hanley and McNeil was used [16]. Furthermore, by using crosstabs, we calculated the sensitivity and specificity for the NLCR in diagnosing sepsis and bacteremia at a cut-off value of 10 based on previous studies [14, 17, 18]. Furthermore, we calculated an optimal cut-off value of 10 as well. The normal range for CRP, PCT, WBC count, neutrophil count and lymphocyte count was <50 mg/l, <0.50ng/ml, ≥4.0*10^9^/l and ≤12*10^9^/l, ≤10*10^9^/l and ≥1.0*10^9^/l, respectively. To investigate whether or not the inflammation parameters were correlated with APACHE scores, we divided the patients into quartiles depending on their APACHE score and constructed separate ROC-curves for each quartile.

To evaluate the association between the NLCR and the secondary outcomes, we categorized the patients by quartile of the NLCR. The first quartile was defined as the reference group. We calculated the Odds Ratio (OR) with 95% confidence intervals for categorical variables and the medians for continuous variables. A P value below 0.05 (two-sided) was considered statistically significant.

## Results

### Patients

**Fig 1:**
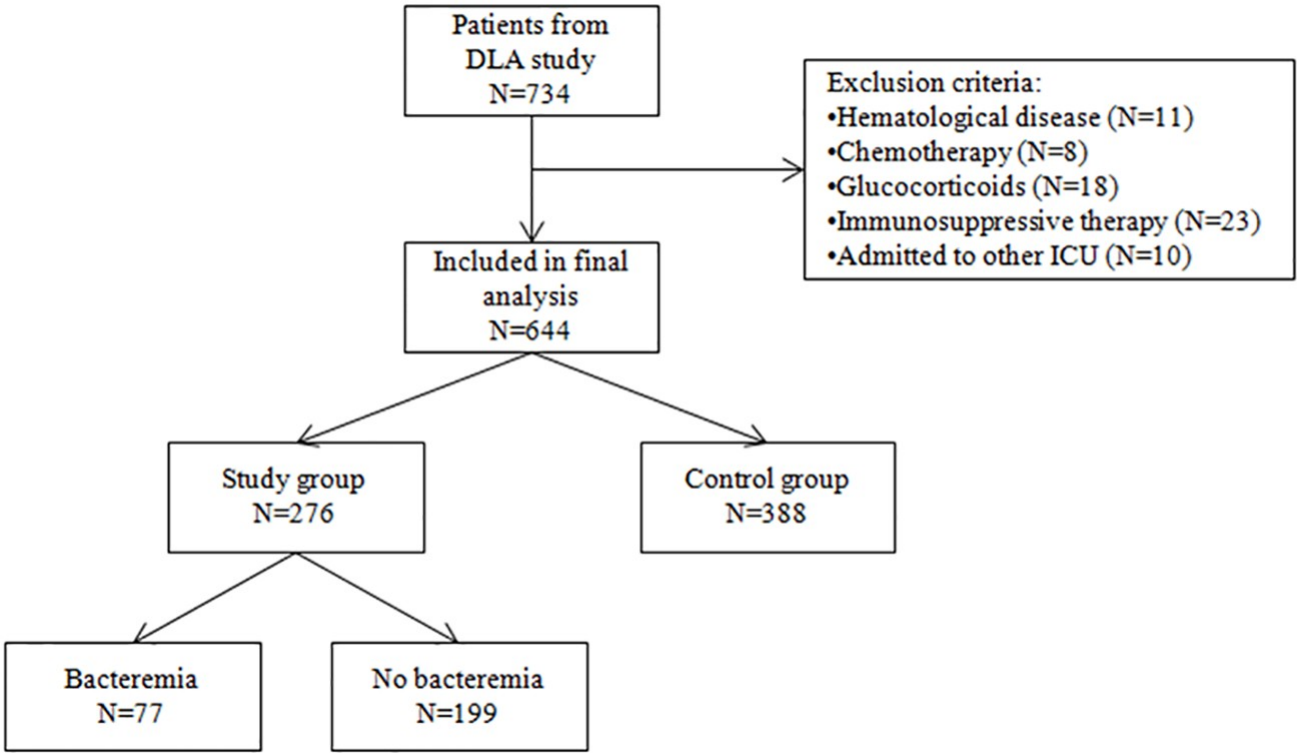
Enrollment flow chart. DLA = Dynamic Light Application.

Of the 734 patients in the DLA study, 70 patients were excluded in the current study because of either a known or probable immune deficiency (N = 60), or admission to an ICU elsewhere before admission to the ICU of the Jeroen Bosch Hospital (N = 10) (Fig 1). A total of 664 patients were included in this study, of whom 276 patients met the criteria for sepsis (41.6%; *sepsis group*) and 388 did not (58.4%; *non-sepsis group*). Patient characteristics are shown in Table 1. There were no significant differences in baseline clinical characteristics between the sepsis and non-sepsis group, except for the number of patients with diabetes In the sepsis group, the most frequent sites of origin of sepsis was pulmonary infection (N = 120; 43.5%) (see S1 Table). Significantly more patients in the sepsis group were treated for at least 24 hours with antibiotics prior to admission to the ICU (P<0.001). In the non-sepsis group, more patients were admitted to the ICU within 24 hours after arrival at the ED (67.8% compared to 52.9%, P<0.001).

**Table 1:** Patient characteristics.

	Sepsis group (N = 276)	Non-sepsis group (N = 388)	P-value
*Male*	156 (56.5%)	234 (60.3%)	0.33
*Age*	66 (±13)	65 (±14)	0.36
*Apache II score*	23 (±8)	22 (±8)	0.17
*Admission diagnosis*			
*Surgical*	75 (27.2%)	89 (22.9%)	0.24
*Medical*	195 (70.7%)	258 (66.5%)	0.27
*Neurological*	6 (2.2%)	12 (3.1%)	0.63
*Trauma*	0 (0.0%)	29 (7.5%)	NA
*Comorbidity*			
*Cardiac insufficiency*	8 (2.9%)	13 (3.4%)	0.74
*Renal failure*	24 (8.7%)	20 (5.2%)	0.07
*Liver cirrhosis*	2 (0.7%)	3 (0.8%)	1.000
*COPD*	14 (5.1%)	21 (5.4%)	0.85
*Diabetes mellitus*	46 (16.7%)	39 (10.1%)	**0.012**
*Neoplasm*	5 (1.8%)	4 (1.0%)	0.50
*Alcohol abuse*	23 (8.3%)	24 (6.2%)	0.29
*Smoking*	97 (35.1%)	133 (34.3%)	0.82
*Previous AB usage*	112 (40.6%)	57 (14.7%)	**<0.001**
*Admission from ED*	146 (52.9%)	263 (67.8%)	**<0.001**

Data presented as number (percentage) or mean (standard deviation). NA = not applicable; COPD = chronic obstructive pulmonary disease; AB = antibiotic; ED = emergency department.

### Sepsis

In the sepsis group, infection was confirmed in 185 patients (67.0%) by microbiological investigation, either by the detection of micro-organisms in blood cultures and/or cultures of other specimens, or by urine immunochromatographic antigen detection, ELISA or PCR. Bacteremia was present in 77 patients (27.9%) with sepsis. Infection was confirmed by radiological investigation and procedure in 204 patients (73.9%) and 87 patients (31.5%), respectively.

### Inflammatory markers

The inflammatory markers at the time of ICU admission of the sepsis and non-sepsis group are shown in Table 2. The inflammatory markers of patients with bacteremia and patients without bacteremia in the sepsis group are shown in Table 3.

**Table 2:** Inflammatory markers in sepsis and non-sepsis group.

	Sepsis group (N = 276)	Non-sepsis group (N = 388)	P value
*CRP*	161 [106–259]	13 [5–56]	**<0.001**
*PCT*	4.60 [0.87–23.75]	0.13 [0.07–0.38]	**<0.001**
*WBC count*	12.7 [7.8–18.6]	12.0 [8.6–16.4]	0.50
*Neutrophil count*	11.4 [6.6–16.8]	9.9 [7.1–14.5]	0.13
*Lymphocyte count*	0.7 [0.4–1.0]	1.1 [0.7–1.5]	**<0.001**
*NLCR*	15.3 [8.5–29.5]	9.3 [6.2–14.5]	**<0.001**

Data presented as median [interquartile range]. CRP = C-reactive protein; PCT = procalcitonin; WBC = white blood cell; NLCR = neutrophil-lymphocyte count ratio

**Table 3:** Inflammatory markers in patients with sepsis.

	Bacteremia (N = 77)	No bacteremia (N = 199)	P value
*CRP*	202 [143–288]	148 [98–246]	**0.001**
*PCT*	16.00 [3.40–45.50]	2.60 [0.58–14.00]	**<0.001**
*WBC count*	13.8 [9.0–20.4]	12.2 [7.4–17.4]	**0.040**
*Neutrophil count*	13.5 [8.2–18.7]	10.9 [6.2–15.2]	**0.018**
*Lymphocyte count*	0.6 [0.4–1.0]	0.7 [0.4–1.0]	0.13
*NLCR*	18.3 [10.8–38.2]	14.5 [7.8–26.5]	**0.001**

Data presented as median [interquartile range]. CRP = C-reactive protein; PCT = procalcitonin; WBC = white blood cell; NLCR = neutrophil-lymphocyte count ratio

The NLCR was significantly higher in patients with sepsis compared to patients without sepsis (median (interquartile range [IQR]) 15.3 [10.8–38.2] versus 9.3 [6.2–14.5]; P<0.001). The NLCR was also significantly different between the sepsis group patients with and without bacteremia (18.3 [10.8–38.2] versus 14.5 [7.8–26.5]; P = 0.001).

Similar differences between patients with sepsis compared to patients without sepsis were found for CRP level, PCT level and lymphocyte counts (see Table 3). Of these markers, CRP level and PCT level were also significantly different within the sepsis group in patients with bacteremia compared to sepsis patients without bacteremia. There was no significant difference in WBC count and neutrophil count between the sepsis and non-sepsis group. However, within the sepsis group, both were significantly higher in patients with bacteremia compared to patients without bacteremia.

**Fig 2:**
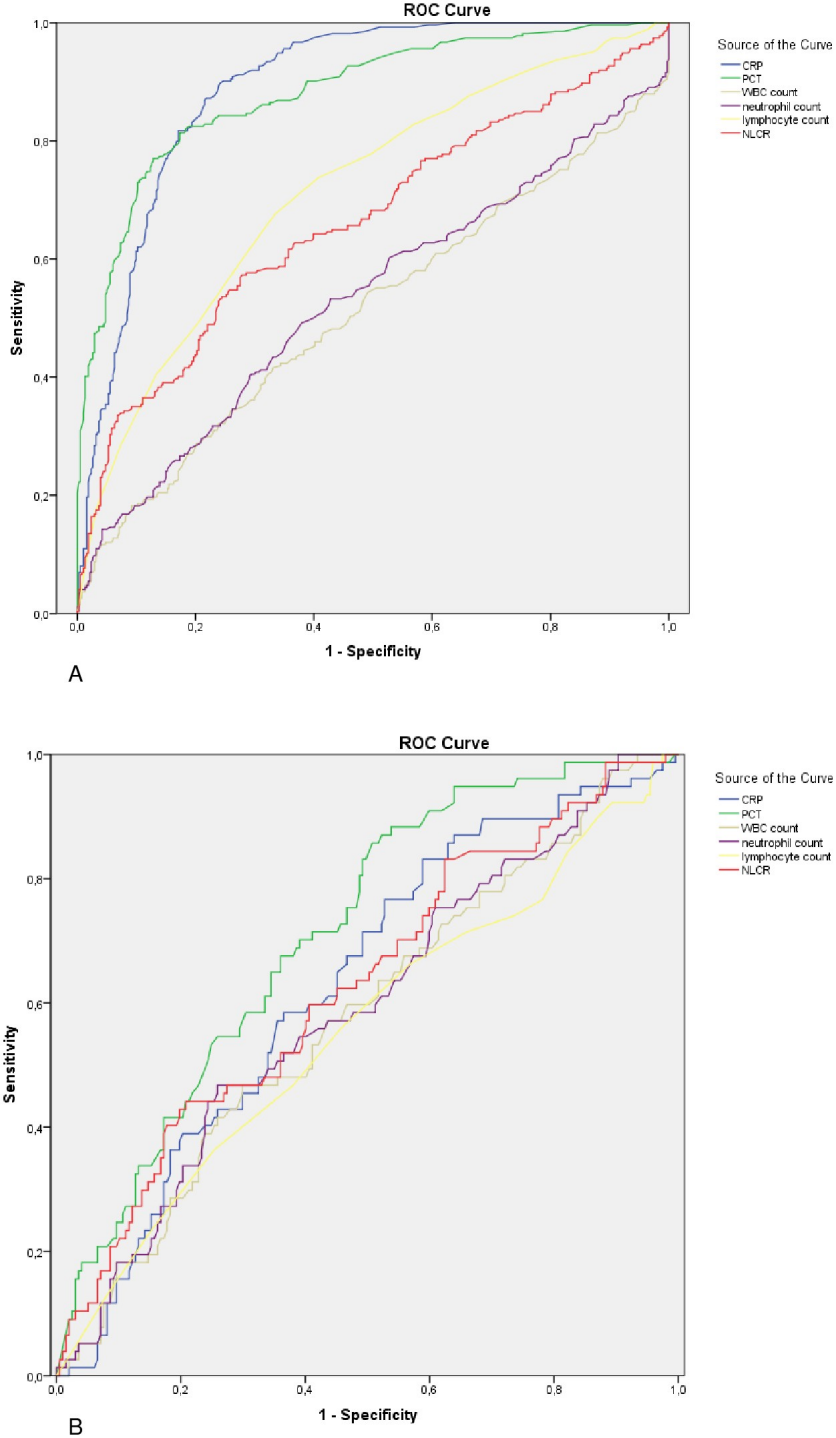
Receiver operating characteristics (ROC) curves of inflammatory markers for differentiating sepsis and bacteremia. (A) ROC curves of inflammatory markers for differentiating sepsis. (B) ROC curves of inflammatory markers for differentiating bacteremia. CRP = C-reactive protein; PCT = procalcitonin; WBC = white blood cell; NLCR = neutrophil-lymphocyte count ratio.

Fig 2 shows ROC curves of all inflammatory markers for differentiating patients with sepsis from patients without sepsis (A) and for differentiating patients with bacteremia from patients without bacteremia in the sepsis group (B). The AUROC curves are shown in Table 4.

**Table 4:** Evaluation of inflammatory markers for detecting sepsis and for diagnosing bacteremia in patients with sepsis.

	Sepsis (N = 664)	Bacteremia (N = 276)
*CRP*	0.89 (0.87–0.92)	0.63 (0.56–0.70)
*PCT*	0.88 (0.86–0.91)	0.71 (0.65–0.78)
*WBC count*	0.51 (0.47–0.56)	0.58 (0.51–0.66)
*Neutrophil count*	0.53 (0.49–0.58)	0.59 (0.52–0.67)
*Lymphocyte count*	0.72 (0.68–0.76)	0.56 (0.48–0.64)
*NLCR*	0.66 (0.62–0.71)*	0.63 (0.55–0.70)†

Data presented as area under the Receiver Operating Characteristics curve (95% Confidence Interval). CRP = C-reactive protein; PCT = procalcitonin; WBC = white blood cell; NLCR = neutrophil-lymphocyte count ratio

*P<0.001 for comparing the NLCR with WBC count, neutrophil count, CRP and PCT.

^†^ No significant differences between the AUROCs in diagnosing bacteremia.

The area under the ROC curve (AUROC) of the NLCR to detect sepsis was 0.66 (95% CI = 0.62–0.71). We calculated an optimal cut-off value of 10, which was also found in previous studies. Using a cut-off value of 10, the sensitivity and specificity of the NLCR for predicting sepsis was 65.7% (95% CI 59.7%-71.3%) and 53.0% (95% CI 47.9%-58.1%), respectively. In the sepsis group, the AUROC of the NLCR to detect bacteremia was 0.63 (95% CI = 0.55–0.70). The sensitivity and specificity of the NLCR to detect bacteremia at a cut-off value of 10 was 76.1% (95% CI 64.5%-85.4%) and 37.9% (95% CI 31.2%-45.0%), respectively.

The AUROC to detect sepsis was highest for CRP level with an AUROC of 0.89 (95% CI 0.87–0.92) and PCT level with an AUROC of 0.88 (95% CI 0.86–0.91). There was no significant difference between the AUROC of CRP level and PCT level (P = 0.653). The AUROC of the NLCR was significantly higher compared to WBC count (P<0.001) and neutrophil count (P<0.001) and was significantly lower compared to CRP level (P<0.001) and PCT level (<0.001).

There was no significant difference between the AUROCs of the inflammatory parameters in diagnosing bacteremia.

When dividing patients into quartiles depending on their APACHE score and constructing separate ROC curves for each quartile, we found no significant difference between each quartile and the entire cohort of patients (S1 Fig).

### Secondary outcomes

The quartiles in the entire study population were defined as follows: first quartile = NLCR below 7.0; second quartile = NLCR 7.0–11.2; third quartile = NLCR 11.2–18.9 and fourth quartile = NLCR above 18.9. The secondary outcome across quartiles of the NLCR are shown in Table 5.

**Table 5:** Mortality across quartiles of NLCR.

Quartile of NLCR	Mortality in ICU	In-hospital mortality	6-month mortality
*Q1 (<7*.*0)*	REF	REF	REF
*Q2 (7*.*0–11*.*2)*	0.900 (0.494–1.638)	0.926 (0.562–1.525)	1.152 (0.762–1.741)
*Q3 (11*.*2–18*.*9)*	1.100 (0.625–1.936)	1.148 (0.719–1.834)	1.273 (0.852–1.901)
*Q4 (>18*.*9)*	1.093 (0.621–1.925)	1.362 (0.872–2.129)	1.476 (1.004–2.169)*

Data presented as Odds Ratio (95% Confidence Interval). NLCR = neutrophil-lymphocyte count ratio; ICU = Intensive Care Unit; Q = quartile; REF = reference

* = statistically significant

The overall mortality in the ICU, in-hospital mortality and 6-month mortality was 12.7%, 18.4% and 24.8%, respectively. Other than the 6-month mortality in the fourth quartile (OR (95%CI) 1.48 (1.00–2.17)), the risk of mortality was similar between quartiles of the NLCR.

The duration of ICU and hospital stay in all patients was 4 days (IQR [2–9]) and 15 days [9–27], respectively. There were no significant differences in the duration of ICU stay and hospitalization across the quartiles of the NLCR. We observed no significant differences between patients with high or low APACHE II scores (S4).

## Discussion

In the current study we investigated the accuracy of the NLCR to detect the presence of sepsis in ICU patients. Although we found significantly higher NLCR values in patients with sepsis compared to patients without sepsis, our results indicate that CRP and PCT level show more accuracy to detect sepsis.

The physiological immune response to stress is characterized by an increase in neutrophil count and a decrease in lymphocyte count [1, 8, 10]. The NLCR has been suggested as an easily obtained inflammatory marker in critically ill patients [11–14]. To our knowledge, no previous studies have investigated the association between the NLCR and the presence of sepsis in ICU patients. There are some studies that are quite similar to our study [13, 14, 17–19]. However, these studies were conducted at the ED or they concentrated on critically ill patients in the ICU with infection only, in which patients without sepsis or without infection were excluded from the study. These studies in patients in the ICU investigated the NLCR in predicting death from sepsis in the ICU, rather than diagnosing sepsis. Regarding the association between the NLCR and bacteremia, the AUROC of the NLCR in the current study was 0.63. This is in contrast with previous studies in non-ICU patients, where higher AUROCs were found [14, 17, 20, 21]. Several reasons may explain our results.

First, in accordance to the sepsis guidelines, the vast majority of patients admitted to the ICU with suspected sepsis were treated with antibiotics by the general practitioner or during their stay in the ED. In the current study, 52.9% of patients were admitted from the ED. All patients who are admitted at the ED with a suspicion of sepsis, are treated with broad-spectrum antibiotics, with or without gentamycin, depending on the severity of illness. Furthermore, 40.6% of patients with sepsis in the ICU were treated with antibiotics for over 24 hours prior to admission to the ICU. A recent study demonstrated that antibiotic treatment significantly reduces the NLCR [18]. In this study, only 8.2% of patients were treated with antibiotics prior to admission to the ED. Since antibiotic treatment significantly reduces the NLCR and significantly more patients in the ICU have received antibiotic treatment prior to admission compared to patients in the ED, this might explain the lower value of the NLCR in predicting bacteremia in the current study in comparison to previous studies, which were conducted at the ED.

Second, the NLCR is not only increased in patients with infection. Previous studies describe an increase in the NLCR in the first few days after surgery or trauma [22, 23]. Furthermore, the NLCR is higher in patients with COPD compared to healthy individuals and also higher during an exacerbation of COPD compared to stable disease [24, 25]. Our control group consisted of many patients who were admitted to the ICU for monitoring following major elective surgery, trauma or an exacerbation of COPD. Therefore, this may have resulted in a lower predictive value of the NLCR in detecting sepsis.

We found remarkably high AUROCs of CRP level and PCT level compared to previous studies [6, 14, 18, 19, 26]. These studies, however, excluded patients without SIRS or suspected infection, or they compared patients with bacteremia to patients without bacteremia. In the current study however, we included all patients admitted to the ICU and divided them into two groups depending on the presence of sepsis. Furthermore, our non-sepsis group consisted of few patients with infection (but without SIRS) and a relatively high number of patients with cardiac arrest. These patients had low CRP levels, in accordance with other studies, thereby enhancing the discriminatory capacity of CRP level [27, 28].

The NLCR was not associated with the length of ICU or hospital stay. Furthermore, although not significant, we found a trend towards increased mortality with increased quartiles of the NLCR, which is in concordance with previous studies [12, 13].

This study has several limitations. First, this was a single-center retrospective cohort study in a mixed medical-surgical ICU with a high number of cardiac arrest patients, which may make the results less generalizable. Second, there are some important differences in our sepsis and non-sepsis group concerning previous antibiotic usage and source of admission. This is due to the heterogeneity of our control group. Previous studies have shown that the NLCR differs in time [29]. Unfortunately, data concerning the duration of illness before admission to the ICU was not collected during the conduct of the Dynamic Light Study. However, with the data available we can draw some conclusions regarding this question. In patients with sepsis, 40.6% of patients were treated with antibiotics for over 24 hours and therefore presumably had symptoms for over 24 hours. In the non-sepsis group, patients who were admitted for cardiac arrest or trauma, accounting for 30.9% of patients in this group, had symptoms for less than 24 hours. For the majority of patients however, the duration of symptoms is not known. Third, sepsis is a clinical diagnosis with a lack of a gold standard test. We defined sepsis as the presence of both SIRS and infection, the most frequently used definition since 1991 [30]. To improve the diagnosis of sepsis, recently new definitions of sepsis and septic shock have been introduced, in which sepsis is defined as a life-threatening organ dysfunction caused by a dysregulated host response to infection [31, 32]. This definition focuses more on organ dysfunction, evaluated using the Sequential (Sepsis-related) Organ Failure Assessment (SOFA) score, rather than the inflammatory host response [33]. However, also in this new definition, sepsis remains a clinical diagnosis. Furthermore, there is a relatively lack of experience with the new sepsis-3 criteria and few studies have been performed using these criteria. In order to be able to compare our results to previous studies, the old sepsis criteria were used in this study. Fourth, we investigated the association between the inflammation parameters and the presence of sepsis with or without bacteremia in only one blood sample, taken within 6 hours prior to or after ICU admission. Riche et al showed that the NLCR differs in time and that early and late death from septic shock are associated with a low and high NLCR, respectively [29]. However, since early diagnosis and treatment of sepsis are of utmost importance in its outcome, we investigated whether or not a diagnosis of sepsis can be established at admission to the ICU. Therefore, we have chosen to use only one blood sample taken at admission to the ICU.

### Conclusion

In this study, evaluating the association between the NLCR and the presence of sepsis in patients admitted to the ICU, we found significantly higher values of the NLCR in patients with sepsis compared to patients without sepsis. However, compared to CRP and PCT, the diagnostic accuracy of the NLCR to detect sepsis in ICU patients in this study is low and therefore the NLCR seems less suitable in predicting presence of sepsis in this vulnerable patient category. Considering the limitations of the current study, larger, prospective trials, including the current definition of sepsis, are necessary to establish the role of the NLCR in diagnosing sepsis in any patient admitted to the ICU more firmly.

## Supporting information

S1 Text Exclusion criteria. Details of the exclusion criteria of the Dynamic Light Application study.
(DOCX)

S1 Table Origin of sepsis in study group. Data presented as number (percentage).
(DOCX)

S2 Table Reason for admission in control group. Data presented as number (percentage).
(DOCX)

S1 Fig Receiver operating characteristics (ROC) curves of inflammatory markers for differentiating sepsis, sorted by first (A), second (B), third (C) and fourth (D) quartile of the APACHE score. CRP = C-reactive protein; PCT = procalcitonin; WBC = white blood cell; NLCR = neutrophil-lymphocyte count ratio.
(TIF)

S1 Dataset Database of study patients. (XLSX)

## References

[1] Annane D, Bellissant E, Cavaillon JM. Septic shock. Lancet. 2005;365(9453):63–78. Epub 2005/01/11. 10.1016/S0140-6736(04)17667-8 . 15639681

[2] Clyne B, Olshaker JS. The C-reactive protein. J Emerg Med. 1999;17(6):1019–25. Epub 1999/12/14. . 10595891 10.1016/s0736-4679(99)00135-3

[3] Luzzani A, Polati E, Dorizzi R, Rungatscher A, Pavan R, Merlini A. Comparison of procalcitonin and C-reactive protein as markers of sepsis. Crit Care Med. 2003;31(6):1737–41. Epub 2003/06/10. 10.1097/01.CCM.0000063440.19188.ED . 12794413

[4] Pierrakos C, Vincent JL. Sepsis biomarkers: a review. Crit Care. 2010;14(1):R15 Epub 2010/02/11. 10.1186/cc8872 . 20144219 PMC2875530

[5] Uzzan B, Cohen R, Nicolas P, Cucherat M, Perret GY. Procalcitonin as a diagnostic test for sepsis in critically ill adults and after surgery or trauma: a systematic review and meta-analysis. Crit Care Med. 2006;34(7):1996–2003. Epub 2006/05/23. 10.1097/01.CCM.0000226413.54364.36 . 16715031

[6] Tang BM, Eslick GD, Craig JC, McLean AS. Accuracy of procalcitonin for sepsis diagnosis in critically ill patients: systematic review and meta-analysis. Lancet Infect Dis. 2007;7(3):210–7. Epub 2007/02/24. 10.1016/S1473-3099(07)70052-X . 17317602

[7] Wacker C, Prkno A, Brunkhorst FM, Schlattmann P. Procalcitonin as a diagnostic marker for sepsis: a systematic review and meta-analysis. Lancet Infect Dis. 2013;13(5):426–35. Epub 2013/02/05. 10.1016/S1473-3099(12)70323-7 . 23375419

[8] Adib-Conquy M, Cavaillon JM. Compensatory anti-inflammatory response syndrome. Thromb Haemost. 2009;101(1):36–47. Epub 2009/01/10. . 19132187

[9] Summers C, Rankin SM, Condliffe AM, Singh N, Peters AM, Chilvers ER. Neutrophil kinetics in health and disease. Trends Immunol. 2010;31(8):318–24. Epub 2010/07/14. 10.1016/j.it.2010.05.006 . 20620114 PMC2930213

[10] Luan YY, Dong N, Xie M, Xiao XZ, Yao YM. The significance and regulatory mechanisms of innate immune cells in the development of sepsis. J Interferon Cytokine Res. 2014;34(1):2–15. Epub 2013/09/07. 10.1089/jir.2013.0042 . 24006870 PMC3887438

[11] Zahorec R. Ratio of neutrophil to lymphocyte counts—rapid and simple parameter of systemic inflammation and stress in critically ill. Bratisl Lek Listy. 2001;102(1):5–14. Epub 2001/11/29. . 11723675

[12] Salciccioli JD, Marshall DC, Pimentel MA, Santos MD, Pollard T, Celi LA, et al The association between the neutrophil-to-lymphocyte ratio and mortality in critical illness: an observational cohort study. Crit Care. 2015;19:13 Epub 2015/01/20. 10.1186/s13054-014-0731-6 . 25598149 PMC4344736

[13] Akilli NB, Yortanli M, Mutlu H, Gunaydin YK, Koylu R, Akca HS, et al Prognostic importance of neutrophil-lymphocyte ratio in critically ill patients: short- and long-term outcomes. Am J Emerg Med. 2014;32(12):1476–80. 10.1016/j.ajem.2014.09.001 Epub Sep 6. 25264245

[14] de Jager CP, van Wijk PT, Mathoera RB, de Jongh-Leuvenink J, van der Poll T, Wever PC. Lymphocytopenia and neutrophil-lymphocyte count ratio predict bacteremia better than conventional infection markers in an emergency care unit. Crit Care. 2010;14(5):R192 10.1186/cc9309 Epub 2010 Oct 29. 21034463 PMC3219299

[15] Simons KS, Laheij RJ, van den Boogaard M, Moviat MA, Paling AJ, Polderman FN, et al Dynamic light application therapy to reduce the incidence and duration of delirium in intensive-care patients: a randomised controlled trial. Lancet Respir Med. 2016 Epub 2016/02/21. 10.1016/s2213-2600(16)00025-4 . 26895652

[16] Hanley JA, McNeil BJ. A method of comparing the areas under receiver operating characteristic curves derived from the same cases. Radiology. 1983;148(3):839–43. 10.1148/radiology.148.3.6878708 6878708

[17] Loonen AJ, de Jager CP, Tosserams J, Kusters R, Hilbink M, Wever PC, et al Biomarkers and molecular analysis to improve bloodstream infection diagnostics in an emergency care unit. PLoS One. 2014;9(1):e87315 10.1371/journal.pone.0087315 eCollection 2014. 24475269 PMC3903623

[18] de Jager CP, Wever PC, Gemen EF, Kusters R, van Gageldonk-Lafeber AB, van der Poll T, et al The neutrophil-lymphocyte count ratio in patients with community-acquired pneumonia. PLoS One. 2012;7(10):e46561 10.1371/journal.pone.0046561 Epub 2012 Oct 1. 23049706 PMC3462173

[19] Garnacho-Montero J, Huici-Moreno MJ, Gutierrez-Pizarraya A, Lopez I, Marquez-Vacaro JA, Macher H, et al Prognostic and diagnostic value of eosinopenia, C-reactive protein, procalcitonin, and circulating cell-free DNA in critically ill patients admitted with suspicion of sepsis. Crit Care. 2014;18(3):R116 10.1186/cc13908 24903083 PMC4229882

[20] Laukemann S, Kasper N, Kulkarni P, Steiner D, Rast AC, Kutz A, et al Can We Reduce Negative Blood Cultures With Clinical Scores and Blood Markers? Results From an Observational Cohort Study. Medicine (Baltimore). 2015;94(49):e2264 Epub 2015/12/15. 10.1097/md.0000000000002264 . 26656373 PMC5008518

[21] Lowsby R, Gomes C, Jarman I, Lisboa P, Nee PA, Vardhan M, et al Neutrophil to lymphocyte count ratio as an early indicator of blood stream infection in the emergency department. Emerg Med J. 2015;32(7):531–4. 10.1136/emermed-2014-204071 Epub 2014 Sep 2. 25183249

[22] O’Mahony JB, Palder SB, Wood JJ, McIrvine A, Rodrick ML, Demling RH, et al Depression of cellular immunity after multiple trauma in the absence of sepsis. J Trauma. 1984;24(10):869–75. Epub 1984/10/01. . 6238173 10.1097/00005373-198410000-00001

[23] Tabuchi Y, Shinka S, Ishida H. The effects of anesthesia and surgery on count and function of neutrophils. J Anesth. 1989;3(2):123–31. Epub 1989/09/01. 10.1007/s0054090030123 . 15236027

[24] Gunay E, Sarinc Ulasli S, Akar O, Ahsen A, Gunay S, Koyuncu T, et al Neutrophil-to-lymphocyte ratio in chronic obstructive pulmonary disease: a retrospective study. Inflammation. 2014;37(2):374–80. Epub 2013/10/01. 10.1007/s10753-013-9749-1 . 24078279

[25] Furutate R, Ishii T, Motegi T, Hattori K, Kusunoki Y, Gemma A, et al The Neutrophil to Lymphocyte Ratio Is Related to Disease Severity and Exacerbation in Patients with Chronic Obstructive Pulmonary Disease. Intern Med. 2016;55(3):223–9. Epub 2016/02/03. 10.2169/internalmedicine.55.5772 . 26831014

[26] Kim SY, Jeong TD, Lee W, Chun S, Min WK. Procalcitonin in the assessment of bacteraemia in emergency department patients: results of a large retrospective study. Ann Clin Biochem. 2015;52(Pt 6):654–9. Epub 2015/01/13. 10.1177/0004563214568685 . 25575698

[27] Ristagno G, Varpula T, Masson S, Greco M, Bottazzi B, Milani V, et al Elevations of inflammatory markers PTX3 and sST2 after resuscitation from cardiac arrest are associated with multiple organ dysfunction syndrome and early death. Clin Chem Lab Med. 2015;53(11):1847–57. Epub 2015/05/23. 10.1515/cclm-2014-1271 . 25993733

[28] Dell’anna AM, Bini Vinotti J, Beumier M, Orbegozo-Cortes D, Donadello K, Scolletta S, et al C-reactive protein levels after cardiac arrest in patients treated with therapeutic hypothermia. Resuscitation. 2014;85(7):932–8. Epub 2014/04/22. 10.1016/j.resuscitation.2014.04.003 . 24746786

[29] Riche F, Gayat E, Barthelemy R, Le Dorze M, Mateo J, Payen D. Reversal of neutrophil-to-lymphocyte count ratio in early versus late death from septic shock. Crit Care. 2015;19:439 10.1186/s13054-015-1144-x 26671018 PMC4699332

[30] Bone RC, Balk RA, Cerra FB, Dellinger RP, Fein AM, Knaus WA, et al Definitions for sepsis and organ failure and guidelines for the use of innovative therapies in sepsis. The ACCP/SCCM Consensus Conference Committee. American College of Chest Physicians/Society of Critical Care Medicine. Chest. 1992;101(6):1644–55. Epub 1992/06/01. . 1303622 10.1378/chest.101.6.1644

[31] Shankar-Hari M, Phillips GS, Levy ML, Seymour CW, Liu VX, Deutschman CS, et al Developing a New Definition and Assessing New Clinical Criteria for Septic Shock: For the Third International Consensus Definitions for Sepsis and Septic Shock (Sepsis-3). JAMA. 2016;315(8):775–87. Epub 2016/02/24. 10.1001/jama.2016.0289 . 26903336 PMC4910392

[32] Singer M, Deutschman CS, Seymour CW, Shankar-Hari M, Annane D, Bauer M, et al The Third International Consensus Definitions for Sepsis and Septic Shock (Sepsis-3). JAMA. 2016;315(8):801–10. Epub 2016/02/24. 10.1001/jama.2016.0287 . 26903338 PMC4968574

[33] Vincent JL, Moreno R, Takala J, Willatts S, De Mendonca A, Bruining H, et al The SOFA (Sepsis-related Organ Failure Assessment) score to describe organ dysfunction/failure. On behalf of the Working Group on Sepsis-Related Problems of the European Society of Intensive Care Medicine. Intensive Care Med. 1996;22(7):707–10. Epub 1996/07/01. . 8844239 10.1007/BF01709751

