# Supplementary material for: The value of the neutrophil-lymphocyte count ratio in the diagnosis of sepsis in patients admitted to the Intensive Care Unit: A retrospective cohort study
Source: PLoS One. 2019 Feb 27;14(2):e0212861. doi: 10.1371/journal.pone.0212861 (PMC6392273; doi:10.1371/journal.pone.0212861)
Supplement: S1 Text — Details of the exclusion criteria of the Dynamic Light Application study. (DOCX) [file pone.0212861.s001.docx]

**S1 Text. Exclusion criteria.** Details of the exclusion criteria of the Dynamic Light Application study.

Exclusion criteria Dynamic Light Application study:

Patients were excluded if the anticipated life expectancy was less than 48 hours of if patients were unable to be assessed for delirium (e.g. severe hearing loss or visual impairment, unable to understand Dutch, or severe mental impairment).

Glucocorticoid treatment:

Patients were excluded if they had received treatment with glucocorticoids, either short-duration high-dose (e.g. >5 days ≥1mg/kg/day) or long-duration low-dose (e.g. >20 days ≥0.1mg/kg/day).
