# Supplementary material for: The value of the neutrophil-lymphocyte count ratio in the diagnosis of sepsis in patients admitted to the Intensive Care Unit: A retrospective cohort study
Source: PLoS One. 2019 Feb 27;14(2):e0212861. doi: 10.1371/journal.pone.0212861 (PMC6392273; doi:10.1371/journal.pone.0212861)
Supplement: S1 Table — Data presented as number (percentage). (DOCX) [file pone.0212861.s002.docx]

**S1 Table. Origin of sepsis in study group.**

| **Origin of sepsis** | **Number (%)** |
| --- | --- |
| *Pulmonary infection* | 120 (43.5) |
| *Abdominal infection* | 79 (28.6) |
| *Urinary tract* | 23 (8.3) |
| *Biliary tract* | 23 (8.3) |
| *Meningitis* | 6 (2.2) |
| *Other* | 25 (9.1) |
| **Total** | **276 (100)** |

Data presented as number (percentage).
