# Supplementary material for: The value of the neutrophil-lymphocyte count ratio in the diagnosis of sepsis in patients admitted to the Intensive Care Unit: A retrospective cohort study
Source: PLoS One. 2019 Feb 27;14(2):e0212861. doi: 10.1371/journal.pone.0212861 (PMC6392273; doi:10.1371/journal.pone.0212861)
Supplement: S2 Table — Data presented as number (percentage). (DOCX) [file pone.0212861.s003.docx]

**S2 Table. Reason for admission in control group.**

| **Reason for admission** | **Number (%)** |
| --- | --- |
| *Cardiac arrest* | 92 (23.7) |
| *Surveillance after surgery* | 82 (21.1) |
| *Respiratory insufficiency* | 79 (20.4) |
| *Hemodynamic instability* | 33 (8.6) |
| *Trauma* | 28 (7.2) |
| *Pulmonary embolism* | 12 (3.1) |
| *Thrombolysis* | 11 (2.8) |
| *Other* | 51 (13.1) |
| **Total** | **388 (100)** |

Data presented as number (percentage).
