# Supplementary figures and images for: The value of the neutrophil-lymphocyte count ratio in the diagnosis of sepsis in patients admitted to the Intensive Care Unit: A retrospective cohort study
Source: PLoS One. 2019 Feb 27;14(2):e0212861. doi: 10.1371/journal.pone.0212861 (PMC6392273; doi:10.1371/journal.pone.0212861)

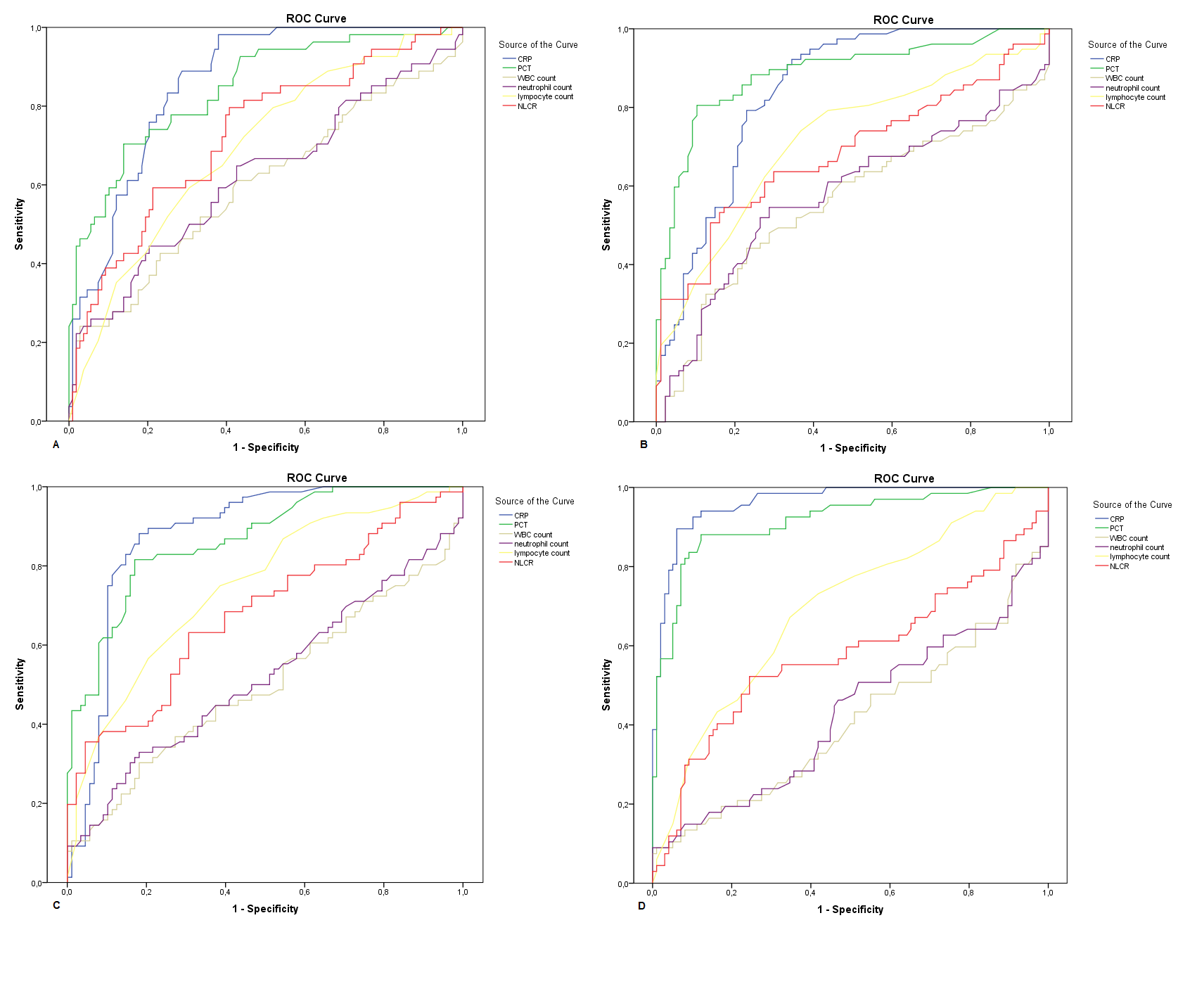

Supplement: S1 Fig — CRP = C-reactive protein; PCT = procalcitonin; WBC = white blood cell; NLCR = neutrophil-lymphocyte count ratio. (TIF) [file pone.0212861.s004.tif]
